# The Role of the WI-38 Cell Strain in Saving Lives and Reducing Morbidity

**DOI:** 10.3934/publichealth.2017.2.127

**Published:** 2017-03-02

**Authors:** S. J. Olshansky, L. Hayflick

**Affiliations:** 1 University of Illinois at Chicago, Chicago, Illinois, USA; 2 University of California at San Francisco, USA

**Keywords:** demography, public health, vaccines, mortality

## Abstract

The modern success story of vaccinations involves a historical chain of events that transformed the discovery that vaccines worked, to administering them to the population. We estimate the number of lives saved and morbidity reduction associated with the discovery of the first human cell strain used for the production of licensed human virus vaccines, known as WI-38. The diseases studied include poliomyelitis, measles, mumps, rubella, varicella (chicken pox), herpes zoster, adenovirus, rabies and Hepatitis A. The number of preventable cases and deaths in the U.S. and across the globe was assessed by holding prevalence rates and disease-specific death rates constant from 1960–2015. Results indicate that the total number of cases of poliomyelitis, measles, mumps, rubella, varicella, adenovirus, rabies and hepatitis A averted or treated with WI-38 related vaccines was 198 million in the U.S. and 4.5 billion globally (720 million in Africa; 387 million in Latin America and the Caribbean; 2.7 billion in Asia; and 455 million in Europe). The total number of deaths averted from these same diseases was approximately 450,000 in the U.S., and 10.3 million globally (1.6 million in Africa; 886 thousand in Latin America and the Caribbean; 6.2 million in Asia; and 1.0 million in Europe).

## 1. Introduction

In late 2014 and early 2015 a measles outbreak occurred in California that began when an unvaccinated 11-year old with an active infection visited a theme park. The disease was subsequently observed in 24 States (not all of which were linked to the California outbreak), with a total of 188 new cases identified in that year [1]. This outbreak was declared over on April 17, 2015. About 88 percent of the earliest cases of measles in California occurred among children who were either unvaccinated, or who had unknown or undocumented vaccine status [2]. The virus type in the California outbreak was identified as B3, which is the same virus type that caused a measles outbreak in the Philippines in 2014, and which has been linked to measles cases in 14 other countries since the California outbreak began.

The Centers for Disease Control and Prevention lists 16 vaccine-preventable diseases—measles among them [3]. Prior to vaccinations in the U.S., 20% of those who developed measles, required hospitalization; poliomyelitis caused about 15,000 cases of paralysis every year; about 85% of infants born to mothers infected with rubella during the first trimester had serious birth defects; and the total estimated morbidity count associated with diseases that are now vaccine preventable was more than 1.1 million cases every year [4]. Although vaccinations have long been known to reduce morbidity and save lives [5], the recent outbreak of measles in the United States has been linked directly to the rise of an anti-vaccination movement [6]. Vaccination rates for measles, mumps and rubella (MMR) in the U.S. are now as low as 50%–86%; well below the threshold of 96%–99% required to achieve herd immunity [7].

If the anti-vaccination movement gains any additional traction, developed and developing nations will have taken a dangerous step backward in protecting public health, especially that of children. There are many ways to re-emphasize the health benefits of vaccinations, but one novel approach that represents a perfect example of applied demography in public health is to illustrate how many lives have been saved, and how many people are alive today, as a result of a single breakthrough in the chain of historical events that led to the development and successful dissemination of live attenuated viral vaccines.^1^ Here we illustrate how the discovery and use of a single cell strain used to grow most viral vaccines in use today (WI-38 [8] and a later derivative [9]), has already had a powerful impact on human life on an order of magnitude that is unprecedented in the history of public health.^2^ This direct application of applied demography will shed new light on (1) the importance of vaccines in saving lives, (2) the chain of fortuitous events that occurred to create a public health breakthrough of this magnitude and, (3) make clear that the anti-vaccination movement represents a serious threat to a proven public health intervention.

A Brief History of Vaccinations

Throughout most of human history, living a long life was rare because communicable diseases killed most people before the age of ten [10]. It wasn't until the discovery and dissemination of public health measures (broadly defined as indoor living and working environments, cooking [which kills pathogenic organisms], hand washing, refrigeration, sewage treatment and waste removal, clean water, and medical interventions (such as vaccinations) that the duration of life attained by many in both developed and developing nations, increased dramatically.

Among the countless critical developments in the history of public health, one of the earliest and most important was the scientific status conferred to vaccination through the work of Edward Jenner in 1796 [11]. Prior to Jenner's discovery that dairymaids exposed to cowpox were immune to smallpox, inoculation (referred to as variolation at the time) was a common practice. This required the inoculator to lance a pustule on someone with active cowpox, and deliver the inoculant subcutaneously to a person previously unexposed. Not everyone benefitted from this procedure and some even died as a result. Jenner's discovery was that exposure to the milder cowpox conferred protection from smallpox - a related but usually lethal communicable disease.

Vaccination is now one of the foundational legs of public health. In the 20^th^ century, the top eight infectious diseases that are now amenable to treatment through viral and bacterial vaccines (smallpox, measles, whooping cough (pertussis), tetanus, meningitis, Hepatitis B, diphtheria and poliomyelitis) accounted for an estimated 600 million deaths and countless more cases of disability. Vaccines in use today are responsible for annually saving an estimated 3 million lives worldwide, and many more are saved from permanent disability. In spite of documented health and longevity benefits of vaccination,^3^ an estimated 1.4 million children under the age of 5 still die every year due to lack of access to vaccines [12].

Critical to the modern success story of vaccinations, was a series of events required to transform the discovery that vaccines worked, to actually developing, testing, and administering them to large segments of the population.^4^ The chain of discovery behind vaccine development includes: (1) isolating the etiological agent that causes a disease (a virus in the example discussed here); (2) developing a method of enabling the virus to reproduce so that sufficient progeny are produced to make a vaccine (viruses are obligate intracellular parasites that are unable to reproduce without a living cell as a host); (3) attenuating or killing the virus so it can no longer produce disease; (4) purifying the vaccine; (5) testing the vaccine for safety and efficacy; (6) storing and transporting it safely (this may require refrigeration); and then (7) distributing it to the population. A break in any one of these links in the chain of events for vaccine development and use, and the entire system fails. The focus of this analysis will be on step # 2 in this chain of events—the development of a live cell strain used to grow viruses.

In the early development of the poliomyelitis vaccines, the first to be prepared in a cell culture, cells isolated from monkey kidneys (and never transferred from the first or primary vessel) were used to grow the viruses. However, it was discovered that these primary cells were often contaminated with dangerous viruses common to monkeys [13]. One contaminant, S.V. 40, was capable of producing tumors in laboratory animals and transforming cultured normal human cells into cancer cells [14]. Other contaminants were either lethal for vaccine workers or could produce pathology [15]. It was recognized in the early 1960s that step #2 in the chain of virus vaccine development was a critical step for creating safe and efficacious vaccines [16],[17].

The first human cell strain used for the production of licensed human virus vaccines, was WI-38 developed by one of us (L.H.) at the Wistar Institute in Philadelphia in 1962. Unlike primary cell cultures, WI-38 is passaged from one vessel to additional vessels ad seriatim, thus producing almost unlimited numbers of cells from a single source for the manufacture of many human virus vaccines. Because a single cell strain can be frozen for indefinite periods of time, WI-38 has been frozen for 55 years, which is the longest period of time that normal human cells have been frozen. Of great importance, and unlike primary cells, WI-38 was exhaustively tested for safety and efficacy before use [18]. Freezing primary cells for testing is impractical. Since the early 1960's, the vast majority of human virus vaccines have been grown in WI-38 or its derivatives,^5^ making its discovery and continued use a critical innovation in the historical chain of events required for vaccine development [19]. Unlike monkey kidney primary cultures, the importance of WI-38 is that (1) it is derived from a single donor, (2) it is free from contaminating viruses, and (3) it can be frozen for indefinite periods of time and tested for safety and efficacy before use in large scale vaccine manufacture [20]. WI-38 was distributed by Dr. Hayflick gratis to the world's human virus vaccine manufacturers.

Here we illustrate the unique contribution of the WI-38 cell strain to all human virus vaccines administered globally since the strains' origin in 1962, and the impact of these vaccinations on global trends in number of deaths averted and fetal deaths avoided due to the use of the WI-38 based Rubella vaccine. This example of applied demographic research is designed to illustrate how a demographic analysis, when applied to cell biology, yields unique insights into the value of public health interventions and the challenges posed by threats to public health that continue to emerge in the modern era.

## 2. Data and Methods

Vaccines for the following virus-based diseases were developed using WI-38: poliomyelitis, measles, mumps, rubella, varicella (chicken pox), herpes zoster,^6^ adenovirus, rabies and Hepatitis A. Reliable estimates of the number of pre-vaccine annual cases and deaths from most of these diseases in the United States have been published [21]. Due to annual variability in the prevalence and death rate from each disease, 1960 was chosen as a single frame of reference for estimating the health impact of the vaccines. Prevalence rates and disease-specific death rates were held constant from 1960 through 2015 as a way to assess the hypothetical impact of vaccine dissemination on vaccine-preventable cases and deaths. That is, it was assumed that prevalence rates and death rates from these diseases observed in 1960 would have prevailed annually through 2015 in the absence of vaccines, thus reflecting the independent effect of growing population size on disease prevalence. Since vaccines were introduced in different years beginning in 1963, only those years from vaccine introduction to 2015 were used in each case (for example, the prevalence rate of poliomyelitis observed in 1960 was 36,110 cases per 3.04 million people in the U.S., see Table 1, this rate was assumed to apply annually to the observed U.S. population from 1963 through 2015). Cases and deaths were then summed between year of vaccine introduction and 2015 as an estimate of the cumulative health impact of the vaccine. Vaccine coverage was assumed to be 95 percent.

## 3. Results

The estimated total number of cases of poliomyelitis, measles, mumps, rubella, varicella, adenovirus, rabies, and hepatitis A averted or treated in the U.S. alone due to the introduction of vaccines developed with the WI-38 cell strain, is 198 million (Table 1). The estimated total number of deaths averted from these same diseases in the U.S. is approximately 450,000. In 1964–65, rubella caused 11,000 fetal deaths in the U.S. [22]. This implies that the rubella vaccine introduced in 1969 averted approximately 633,000 fetal deaths in the U.S. since it was first introduced.

**Table 1.** Viral diseases treated with vaccines prepared using the WI-38 cell strain or its derivatives, year each vaccine was introduced, annual cases, pre-vaccine annual deaths, and cases and deaths averted or treated from each disease from year of introduction to 2015 (with 95% coverage).

Disease	YearIntroduced	Vaccineannual cases(U.S., 1960)	Pre-vaccineannual deaths(U.S.)	Cases averted or treated with 95% coverage	Deaths averted with 95% coverage
Poliomyelitis	1963	36,110	5,865	2,547,045	413,692
Measles	1969–70	530,217	440	34,137,129	28,329
Mumps	1967	162,344	39	10,792,317	2,593
Rubella	1969	47,745	17	3,073,981	1,095
Varicella (chicken pox)	1995–96	4,085,120	107	133,691,807	3,436
Hepatitis A	1996	117,333	137	3,674,988	4,291
Rabies#	1974	18,000	-	10,000,000	-
Adenovirus*	1964	11,138	-	375,619	-
Total (U.S.)		5,017,007	6,603	198,292,887	453,435

Estimates of cases and deaths were obtained from the following sources:

http://www.cdc.gov/vaccines/pubs/pinkbook/downloads/appendices/G/cases-deaths.pdf

Roush SW, Murphy TV (2007) *JAMA* 298: 2155-2163.

http://jama.jamanetwork.com/article.aspx?articleid=209448.

Population estimates were obtained from the following sources:

http://www.census.gov/population/international/data/worldpop/table_population.php.

http://www.census.gov/prod/cen1990/cph2/cph-2-1-1.pdf.

# The majority of cases of rabies treated with the WI-38 based vaccine occurred after the disease appeared rather than as a preventative measure (http://www.rightdiagnosis.com/r/rabies/stats.htm). The value of the WI-38 based rabies vaccine was that it eliminated the painful side effects associated with the previous vaccine made in neuronal tissue.

* The adenovirus was developed in 1966 and first administered to military personnel in 1971. Estimates provided here assume .04% of the total U.S. population served in the armed forces annually; prevalence of adenovirus among military forces is 1.1% annually; and these calculations apply only for the years 1971–1999 and 2011–2016 when the WI-38 related adenovirus vaccine was administered.

Although it is not possible to generate precise global estimates of cases and deaths averted due to the use of vaccines based exclusively on the WI-38 cell strain or its derivatives, a rough approximation may be obtained by assuming (very conservatively)^7^ that the prevalence rates and death rates for these diseases observed in the U.S. apply equally to the entire human population. Under this assumption, the estimated total number of cases of poliomyelitis, measles, mumps, rubella, varicella, adenovirus, rabies and hepatitis A averted or treated due to the introduction of vaccines developed with the WI-38 cell strain and its derivatives, is about 4.5 billion globally (720 million in Africa; 387 million in Latin America and the Caribbean; 2.7 billion in Asia; and 455 million in Europe).^8^ The estimated total number of deaths averted from these same diseases is about 10.3 million (1.6 million in Africa; 886 thousand in Latin America and the Caribbean; 6.2 million in Asia; and 1.0 million in Europe).

## 4. Discussion

The history of public health is filled with success stories, but each success has met with significant challenges. In the case of Edward Jenner's work on a vaccine for smallpox, he was first attacked and ridiculed for his ideas and, of course, later vindicated. The modern rise of the anti-vaccine movement accelerated with a manuscript published in 1998 claiming that the MMR vaccine *caused* developmental disorders in children [23]. Even though that article was retracted by the journal that published it [24], evidence suggests that a significant and dangerously high percentage of the U.S. population either delays or refuses vaccinations for their children within the first 24 months of life [25].

The fact remains that humanity now experiences longer and healthier lives than at any time in history, in large measure, because of the development and dissemination of vaccines that ***prevent*** most of the fatal and disabling communicable diseases that plagued our species for millennia. There is no medication, lifestyle change, public health innovation, or medical procedure ever developed that has even come close to the life-saving, life-extending, and primary prevention benefits associated with vaccines. The initial primary beneficiaries of vaccine development and dissemination have been children, and these benefits have accrued for every generation since vaccines first became widely available. In fact, as those saved from dying early in life live into their working years, national economies also benefit as linkages between the health and wealth of nations has been well established [26].

Each discovery or breakthrough in the chain of events that led to vaccines becoming a public health success story may have occurred eventually. However, timing is important, and there is no question that when the WI-38 cell strain became available in 1962, it was fortuitously discovered at the same time that the primary monkey kidney cells used to manufacture the poliomyelitis vaccines were found to have been contaminated. Thus, the use of WI-38 represented a catalyst for subsequent vaccine development. In fact, the success of the research that resulted in the development of WI-38 in 1962 occurred when federal research funds were not prohibited for use in studying the biology of tissue derived from aborted human fetuses. However, during several subsequent presidential administrations, that prohibition was enforced. If that prohibition had been in effect in 1962, it is unlikely that in the subsequent 55 years, there would be billions of people who benefitted from virus vaccines produced in WI-38.

A delay of even one year in the development of an uncontaminated cell strain for vaccine development would have cost humanity millions of lives and countless more cases of vaccine preventable morbidity. Today, a majority of the world's 7.5 billion people have been vaccinated against viral diseases with the use of the WI-38 cell strain and its derivatives. Nearly everyone born in the developed world since 1962 received at least one vaccine manufactured with the WI-38 cell strain, along with a growing proportion of the population in developing nations. WI-38 and its derivatives are still in use for producing many viral vaccines that are distributed worldwide today.

Billions of people are alive today who would otherwise have either died in childhood or who would have been crippled or disabled by vaccine preventable diseases. The World Health Organization estimates that all immunizations now available avert about 2.5 million deaths among children every year, but many more lives could still be saved if vaccines were universally available. In fact, it is ironic that the rubella vaccine (which is produced in theWI-38 cell strain that originated from an aborted human fetus) is vigorously opposed by anti-choice advocates, even though this vaccine prevented over 633,000 miscarriages in the U.S. alone, and countless more across the globe, and it has prevented tens of millions of clinical health issues in children (e.g., encephalitis, autism, deafness, diabetes, etc.) linked to congenital rubella syndrome [27].

Given the extent to which vaccines were made available to today's generation of reproducing adults when they were children, *it is likely that most of the people involved in today's anti-vaccination movement have improved health (or are alive today) because their parents and pediatricians had the foresight to administer vaccines to them when they were young*. The same health benefits are being denied to their children for reasons that are indefensible from a public health perspective. The anti-vaccination movement endangers the health of an entire generation of children. Its potential to negatively influence national economies is evident, and it threatens a 150-year old public health success story. The signing of Senate Bill No. 277 (Public Health: Vaccinations) by Governor Brown in California strikes the state's personal exemption for immunizations that was being used by some to allow their children to attend school without being vaccinated [28]. This represents a powerful new reaffirmation that vaccinations save lives.

It is possible that the anti-vaccination movement has arisen among younger generations, in part, because they cannot bear witness to the tragedy of disfigurement, morbidity, and death caused by viral and bacterial diseases. However, as the 2015 outbreak of measles in California reminds us, the diseases our ancestors feared so much have not gone away—they lay dormant in many parts of the world where they resurface on occasion as a constant reminder of their existence. They will return if we lower our guard and allow herd immunity to drop below threshold levels. So as a potent reminder of their devastating impact, we provide images of what poliomyelitis, measles, and smallpox (three examples among many) does to human bodies. The anti-vaccination movement is a wake-up call to reinforce defenses against the diseases that plagued humanity from the beginning.

**Figure 1.**
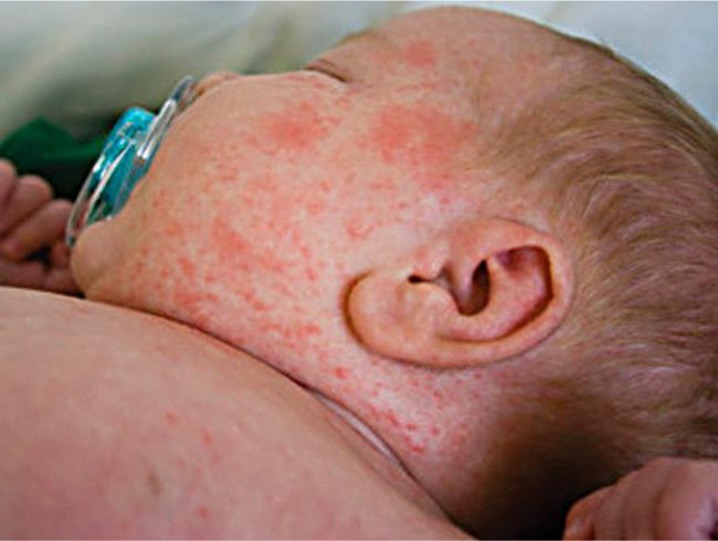
Measles.

**Figure 2.**
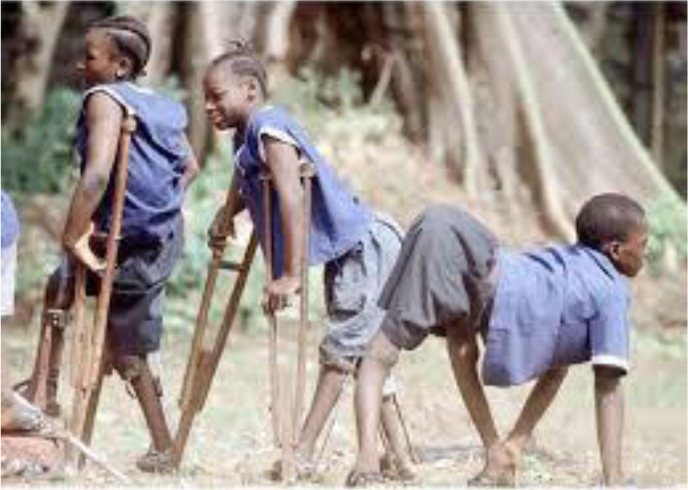
Poliomyelitis.

**Figure 3.**
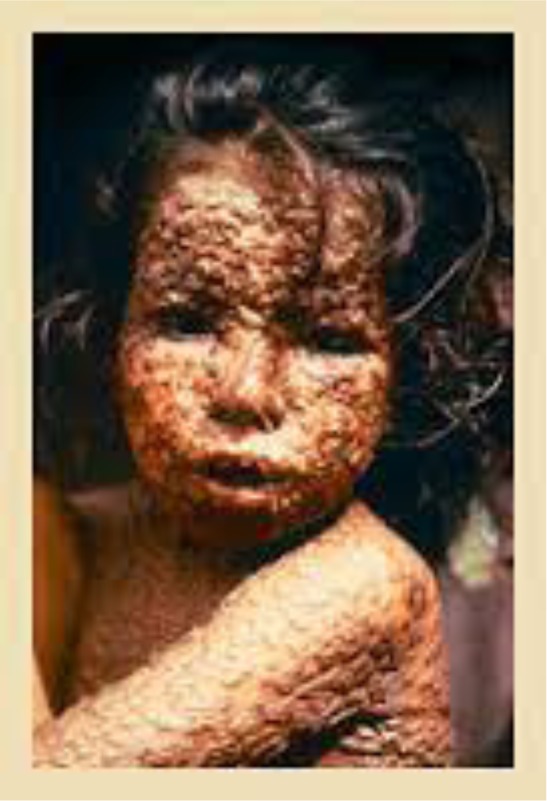
Smallpox.

## Footnotes

1 Our focus in this manuscript is only on live attenuated viral vaccines because we're using the development of a single cell strain (WI-38) critical in their creation as a unique way to illustrate the global health impacts of vaccines in general. This paper is not intended to serve as a review of all vaccine production techniques (such as DNA or recombinant methods), which are acknowledged to be critically important links in the chain of events that led to the successful creation and dissemination of vaccines.

2 We use WI-38 as a point of reference because of its specific link to certain vaccines early in the vaccine movement, and because its development in the early 1960's served as a catalyst for the field. Full credit for the life-saving effects of vaccines belongs to the breakthroughs, scientific advances, and hard work of countless scientists and health care providers, all of whom together contributed to building the chain of vaccine development and use.

3 As an example of what happens when vaccination stops, consider that in 1974 about 80% of Japanese children received pertussis (whooping cough) vaccine. That year there were only 393 cases of whooping cough in the entire country, and not a single pertussis-related death. Then immunization rates for children dropped to 10 percent. In 1979, more than 13,000 people had whooping cough and 41 died. When routine vaccination was resumed, the disease numbers dropped again (http://www.cdc.gov/vaccines/vac-gen/whatifstop.htm#final).

4 The focus of this analysis will be on human virus vaccines only, although it is acknowledged that bacterial vaccines for typhoid, tetanus, pertussis, pneumonia and other bacterial diseases have had a large impact on saving and extending lives.

5 It is important to acknowledge that other uncontaminated human cell strains were subsequently developed. These were established years after the original development of WI-38 was described and used to develop and produce vaccines (e.g, the MRC-5 cell strain was developed in 1970 in the U.K). We consider WI-38 and MRC-5 together in this analysis because they were both important in the historical developments of virus vaccines for public health.

6 Herpes zoster will not be included in this analysis because of complications associated with assessing its prevalence and the effectiveness of the WI-38 based vaccine.

7 The true number of cases and deaths averted is likely to be orders of magnitude greater than this because prevalence and death rates from these diseases are typically higher in developing nations, but these numbers are sufficient to illustrate the minimum health impact of these vaccines.

8 Estimated by taking the fractional proportion of the total population from each of these regions from population estimates provided by the Population Reference Bureau (prb.org), and assuming that the prevalence rates and death rates for these diseases observed in the U.S., apply equally to the entire human population. While it is possible to use the fractional proportion of the total population contained within each of these regions annually since the early 1960s for such an estimate, we do not want to give a false sense of precision by doing so. As noted above, the prevalence and death rates from the U.S. are likely to be lower than that observed in developing nations where most of the global population resides, so the estimates provided here are likely to be very conservative. While all nations are represented in the global estimates, not all nations are represented by the regional data provided here.
